# Evidence of Invasive Reptiles as Novel Paratenic Hosts of *Angiostrongylus cantonensis* in the Canary Islands: Implications for Parasite Transmission in Island Ecosystems

**DOI:** 10.3390/ani16193011

**Published:** 2026-09-24

**Authors:** Nestor Abreu-Acosta, Román Pino-Vera, Natalia Martin-Carrillo, Pilar Foronda

**Affiliations:** 1 Instituto Universitario de Enfermedades Tropicales y Salud Pública de Canarias, Universidad de La Laguna (ULL), Av. Astrofísico F. Sánchez, sn, 38203 La Laguna, Spain; nabreu@ull.edu.es (N.A.-A.); rpinover@ull.edu.es (R.P.-V.); nmartinc@ull.edu.es (N.M.-C.); 2 Departamento de Obstetricia y Ginecología, Pediatría, Medicina Preventiva y Salud Pública, Toxicología, Medicina Legal y Forense y Parasitología, Facultad de Farmacia, Universidad de La Laguna (ULL), Av. Astrofísico F. Sánchez, sn, 38203 La Laguna, Spain

**Keywords:** rat lungworm, *Lampropeltis californiae*, *Anolis porcatus*, one health, zoonosis, biological invasions

## Abstract

*Angiostrongylus cantonensis* is an emerging zoonotic nematode whose transmission relies on complex host interactions. Although reptiles are recognized as paratenic hosts, data on invasive reptilian species remain scarce. This study investigated *A. cantonensis* occurrence in 167 invasive reptiles from the Canary Islands- 51 California kingsnakes (*Lampropeltis californiae*), 74 Cuban green anoles (*Anolis porcatus*), and 42 veiled chameleons (*Chamaeleo calyptratus*)- using nested PCR targeting the internal transcribed spacer 1 (ITS1) region, followed by Sanger sequencing. Parasite DNA was detected in 94.1% of California kingsnakes and 21.6% of Cuban green anoles, whereas all chameleons tested negative. Infection prevalence was significantly higher in California kingsnakes. Neither body size nor sex was associated with infection. These findings provide the first evidence that California kingsnakes and Cuban green anoles act as natural paratenic hosts for *A. cantonensis* in Europe. The high prevalence in the invasive California kingsnake suggests a key role in amplifying parasite transmission within insular ecosystems, emphasizing the need to incorporate invasive reptiles into One Health surveillance programs to mitigate zoonotic risks and protect biodiversity.

## Simple Summary

The rat lungworm is a parasitic worm that can cause severe disease in humans and animals, and its spread depends on various creatures that help the parasite move. While researchers know that reptiles can carry this parasite, very little is known about how invasive reptiles—animals introduced into ecosystems where they do not naturally belong—might spread it. This study aimed to determine whether three invasive reptile species living in the Canary Islands carry this dangerous parasite. By testing 167 animals, we discovered that an alarming 94.1% of the invasive California kingsnakes and 21.6% of the Cuban green anoles carried the parasite’s genetic material, while the chameleons tested negative. These findings provide the first proof in Europe that these two invasive reptiles act as natural carriers of the worm. Because kingsnakes showed such a high infection rate, they likely play a major role in spreading the parasite further across the islands. Recognizing this threat is valuable for society because it highlights the urgent need to include invasive wildlife in public health and environmental monitoring programs to protect local biodiversity and prevent human disease.

## 1. Introduction

*Angiostrongylus cantonensis* (Chen, 1935), commonly known as the rat lungworm, is an emerging zoonotic metastrongyloid nematode with a broad and expanding geographical distribution. Its life cycle involves rats, mainly *Rattus rattus* (Linnaeus, 1758) and *Rattus norvegicus* (Berkenhouth, 1769), as definitive hosts, shedding first-stage larvae (L1) in their feces. Terrestrial and freshwater gastropods act as intermediate hosts, where the L1 larvae undergo two moults (L1-L2-L3) to become infective third-stage (L3) larvae [1,2,3,4,5]. Human infection can cause eosinophilic meningoencephalitis and, in severe cases, neurological sequelae or death [4,6].

Paratenic hosts play an important role in the transmission ecology of *A. cantonensis*. In these hosts, L3 larvae do not develop into adults but can remain viable within tissues and are transmitted to predators through trophic interactions, thereby increasing the likelihood of exposure to higher vertebrates [7]. Recent reviews have documented a wide range of natural and experimental paratenic hosts, including crustaceans, fish, amphibians, reptiles, and terrestrial planarians, highlighting that reptiles may be particularly relevant because they can acquire infection by consuming infected gastropods or other infected prey and may subsequently contribute to trophic transmission of the parasite [7,8]. The public health relevance of this transmission pathway is supported by human cases associated with reptile consumption, where infective larvae are believed to accumulate through trophic biomagnification [9,10]. Although reptile consumption is not practiced in the Canary Islands, the ecological mechanisms of trophic transfer and long-term tissue persistence remain directly applicable for assessing ecological and potential zoonotic risks in insular ecosystems [8].

Island ecosystems are particularly vulnerable to pathogen establishment because of their high endemism, limited functional redundancy, and susceptibility to biological invasions. As *A. cantonensis* transmission is strongly influenced by trophic interactions, introduced species may alter transmission pathways and facilitate parasite persistence. In this context, paratenic hosts such as reptiles may act as mobile tissue reservoirs, potentially contributing to parasite maintenance even when infections in gastropods remain spatially restricted [3,11].

In the Canary Islands, *A. cantonensis* was first reported in 2010 in rats from Tenerife [12]. Subsequent studies have shown that the known distribution of the parasite has expanded with complex spatial distribution patterns [3,13]. Suitable intermediate hosts, including endemic and synanthropic gastropod species such as *Plutonia lamarckii*, *Cornu aspersum* and *Theba pisana,* have also been identified in Tenerife [14]. Importantly, the endemic lizard *Gallotia galloti* (Dumeril and Bibron, 1839) was identified as a natural paratenic host on Tenerife through histopathological and molecular evidence [15], and has been proposed as a sentinel species for monitoring the distribution of the parasite across the island [11].

At the same time, several invasive reptile species have established populations in the archipelago. The California kingsnake (*Lampropeltis californiae*) (Blainville, 1835) is established on Gran Canaria, where it has caused severe declines in endemic reptile populations [16,17]. Owing to its generalist diet and position as a high-level predator, it may accumulate parasites through predation on mollusks and other paratenic reptiles *via* intraguild predation [17,18]. The veiled chameleon (*Chamaeleo calyptratus*) (Duméril and Bibron, 1851) has also established feral populations in Gran Canaria [19], while the Cuban green anole (*Anolis porcatus*) (Gray, 1840) is established in Tenerife [20]. Previous studies have documented ecological characteristics and the carriage of other pathogens in these invasive reptiles, but their potential involvement in the transmission cycle of *A. cantonensis* remains unknown.

Given the documented presence of suitable intermediate hosts, infected rats and a natural paratenic role for endemic lizards in the Canary Islands [13,14,15], these invasive reptiles represent potential additional hosts within the local transmission network.

Therefore, this study aimed to evaluate the potential role of three invasive reptile species in the Canary Islands, California kingsnakes, Cuban green anoles, and veiled chameleons, as paratenic hosts of *A. cantonensis*. To achieve this goal, liver tissues were analyzed using a nested PCR assay targeting the ITS1 region, and infection prevalence was analyzed in relation to host demographic and biometric parameters. By combining molecular detection with host demographic and biometric data, this study aims to improve understanding of the *A. cantonensis* transmission cycle in recently colonized territories, provide evidence to assess risks to insular biodiversity, and contribute to evaluation of zoonotic threats the wildlife—human interface.

## 2. Materials and Methods

### 2.1. Samples Collection

From January 2022 to September 2023, a total of 167 liver samples were examined, including 51 California kingsnakes, 74 Cuban green anoles, and 42 veiled chameleons. The reptiles were sexed following morphological characters, and body weight and length (SVL for Cuban green anoles and veiled chameleons and total length for California kingsnakes) were taken before the necropsy. Of the 51 California kingsnakes, 34 were male, 12 were female, and the sex of five adult individuals could not be determined. These snakes originated from different regions of Gran Canaria, including the eastern (*n* = 31), northeastern (*n* = 6), central (*n* = 4), northwestern (*n* = 7), southeastern (*n* = 1), and southwestern (*n* = 2) parts of the island. A total of 74 Cuban green anoles were analyzed, including 25 males, 45 females, and four juvenile individuals of undetermined sex. All anoles were captured in southern Tenerife. Among the veiled chameleons, 42 individuals were examined, comprising 11 males, 21 females, and 10 individuals of undetermined sex. All chameleons were collected in the municipality of Arucas (Gran Canaria) (Figure 1).

Liver tissues were selected as the target organ. Although recent data indicate that primary larval accumulation sites can vary among reptile species (e.g., skeletal muscle or intestinal wall), the liver consistently remains a major site of larval migration and granuloma formation [8]. Targeting this organ maintains methodological consistency with previous epidemiological studies in the Canary Islands [15,21].

### 2.2. Ethical Statement

All animals were provided by personnel from GESPLAN (Gestión y Planeamiento Territorial y Medioambiental) and RedEXOS (Canary Islands Early Warning Network for the Detection and Control of Invasive Alien Species) and had been euthanized as part of the invasive species eradication program approved by the Government of the Canary Islands. No live animals were used or euthanized specifically for the purpose of this study.

### 2.3. DNA Isolation and PCR Amplification

Liver samples were aseptically collected during necropsy, and approximately 25 mg from each specimen was subjected to genomic DNA extraction following the protocol described by López et al. [22]. Detection of *A. cantonensis* DNA was performed by nested PCR. The first-round PCR used the primers described by Qvarnstrom et al. [23] to amplify the complete internal transcribed spacer 1 (ITS1) region, whereas the second-round PCR employed *A. cantonensis* specific primers as described by Izquierdo-Rodríguez et al. [24] (Table 1). Both PCR reactions were carried out in a final volume of 25 μL containing 1× PCR buffer, 0.2 mM of each dNTP, 1.5 mM MgCl_2_, and 20–40 ng of total genomic DNA per sample. Positive controls (*A. cantonensis* genomic DNA) and negative controls (H_2_O) were included in every assay to verify the performance of the nested PCR.

Following amplification, 5 μL of each PCR product was analyzed by agarose gel electrophoresis (Fisher Bioreagents, Madrid, Spain). Amplicon sizes were estimated by comparison with molecular weight markers (GeneRuler 50 bp DNA Ladder, Thermo Scientific, Vilnius, Lithuania, and HyperLadder 50 bp, Bioline, London, UK). Gels were stained with Real-Safe (Durviz SL, Valencia, Spain), and amplification products were visualized using a ChemiDoc™ XRS+ imaging system (Bio-Rad, Hercules, CA, USA).

### 2.4. Sequencing and Sequence Analysis

Selected amplicons of the expected size (642 bp) were purified using the UltraClean PCR Clean-up Kit (MO BIO Laboratories, Inc., Carlsbad, CA, USA) according to the manufacturer’s instructions. Bidirectional sequencing was performed at Macrogen (Madrid, Spain) using the ITS1 Canto F3 and ITS1 Canto R1 primers [24]. The resulting sequences were edited and manually inspected using MEGA X v2.16.0 (Molecular Evolutionary Genetics Analysis, Hachioji, Japan) software [25]. Sequence identity was subsequently assessed by comparison with reference sequences available in GenBank using the BLAST v2.16.0 nucleotide search tool (BLASTn).

### 2.5. Statistical Analysis

Differences in prevalence among reptile species and sexes were evaluated using the chi-square, Fisher’s exact test was used for pairwise comparisons when expected cell frequencies were low. Differences in body length and body weight between infected and non-infected individuals were evaluated using the Mann–Whitney U test. Exact tests were used when appropriate because of small and/or highly unbalanced group sizes. Statistical significance was established at *p* < 0.05. All statistical analyses were performed using IBM SPSS Statistics software V.29.0.1.0 for Windows (IBM Corporation, Armonk, NY, USA).

During the preparation of this manuscript, the authors used the OpenAI image generation tool integrated into ChatGPT 5.6 for the purpose of creating the graphical abstract based on the content of the manuscript. The prompts used included instructions to create a scientific graphical abstract illustrating the study of invasive reptiles as novel paratenic hosts of *A. cantonensis* in the Canary Islands, including the study species, sampling, parasite detection by nested PCR and Sanger sequencing, prevalence results, and the main implications of the study. A subsequent prompt was used to simplify the graphical abstract and reduce the amount of text while retaining the main scientific information. No specific software version information was provided by the tool. The authors have reviewed and edited the AI-generated output and take full responsibility for the content of this publication.

## 3. Results

### 3.1. Prevalence and Biometric Associations in Lampropeltis californiae, Anolis porcatus and Chamaeleo calyptratus

Among the 51 California kingsnakes examined, the expected amplification product for *A. cantonensis* (642 bp) was detected in the livers of 47 individuals (48/51, 94.1%) (Figure 2). For Cuban green anoles, it was detected in 16 of the 74 liver samples analyzed (21.6%) (Figure 3). However, no *A. cantonensis* DNA was detected in any of the 42 liver samples obtained from the veiled chameleons. BLAST analysis of the sequences obtained from both California kingsnakes, and Cuban green anoles showed 100% sequence identity with *A. cantonensis* (Query Cover = 100%). The nucleotide sequences generated in this study have been deposited in GenBank under accession numbers PX657145–PX657161.

According to sex, no significant differences in prevalence were observed between males and females (Fisher’s exact test, *p* = 1.000) for California kingsnakes (Table 2). Similarly, no significant differences in prevalence were detected between males and females (Fisher’s exact test, *p* = 1.000), males and juveniles (*p* = 0.553), or females and juveniles (*p* = 0.569) for anoles (Table 2).

Infected snakes had a mean total body length of 86.65 ± 20.2 cm (*n* = 31), whereas non-infected individuals had a mean length of 79.50 ± 21.2 cm (*n* = 3). Mean body weight was 219.46 ± 115.09 g in infected individuals and 250.45 ± 182.58 g in non-infected individuals. Neither body length (U = 39.0; *p* = 0.688) nor body weight (U = 46.0; *p* = 1.000) was significantly associated with *A. cantonensis* infection.

For anoles, no significant differences were observed in body length between infected (mean = 6.32 ± 0.63 cm) and non-infected individuals (mean = 6.49 ± 0.99 cm) (U = 439; *p* = 0.75). Likewise, no statistically significant differences in body weight were found between infected (mean = 5.37 ± 1.31 g) and non-infected individuals (mean = 5.60 ± 2.33 g) (U = 505; *p* = 0.598), suggesting that neither length nor body weight were associated with *A. cantonensis* infection.

### 3.2. Interspecific Comparison of Infection

Comparison among reptile species revealed that the prevalence of *A. cantonensis* in California kingsnakes (94.1%) was significantly higher than that recorded in Cuban green anoles (21.6%) (χ^2^ = 45.85; *p* = 1.28 × 10^−11^) and veiled chameleons (0%) (Fisher’s exact test; *p* = 3.12 × 10^−18^). Likewise, the prevalence in Cuban green anoles was significantly higher than that observed in veiled chameleons (Fisher’s exact test; *p* = 0.000491). Sex-specific comparisons showed that female California kingsnakes exhibited a significantly higher prevalence (91.7%) than female Cuban green anoles (24.4%) (χ^2^ = 19.63; *p* = 9.37 × 10^−6^). Similarly, prevalence among male California kingsnakes (94.1%) was significantly higher than among male Cuban green anoles (24.0%) (χ^2^ = 30.9; *p* = 2.72 × 10^−8^) (Table 3).

## 4. Discussion

The present study provides the first evidence of the involvement of invasive terrestrial reptiles in the transmission network of *A. cantonensis* in the Canary Islands, expanding the spectrum of known paratenic hosts in European island ecosystems. In the Canary Islands, the known distribution of *A. cantonensis* appears to be expanding. The parasite was initially detected in the northern part of Tenerife, in areas with a more humid and cool climate, but has more recently been reported on two neighbouring islands. This expanding distribution, together with the identification of new paratenic hosts, suggests that *A. cantonensis* is exploiting an increasingly diverse range of hosts within the island ecosystems [3,13,26]. The detection of the parasite in California kingsnakes and Cuban green anoles is consistent with the heteroxenous life cycle of *A. cantonensis*, in which reptiles, amphibians, and other vertebrates act as paratenic hosts capable of harboring infective L3 larvae within their tissues [10]. Previous detection of *A. cantonensis* in the endemic lizard *G. galloti* on Tenerife had already demonstrated that reptiles can function as paratenic hosts in the Canary Islands [24]. One of the major contributions of the present study is therefore the identification of California kingsnakes and Cuban green anoles as new natural paratenic hosts of *A. cantonensis*. The remarkable diversity of paratenic hosts reported worldwide reflects the ecological plasticity of this parasite and its ability to exploit different trophic networks [7]. Paratenesis, defined as transmission from one paratenic host to another, represents a key ecological adaptation in the life cycle of this nematode and other metastrongylids infecting carnivorous mammals, which rarely consume gastropods directly but frequently prey on rodents, reptiles, and other vertebrates [27]. Consequently, the detection of *A. cantonensis* in California kingsnakes and Cuban green anoles provides new evidence of the parasite ability to exploit invasive reptiles within insular ecosystems and expand the range of paratenic hosts involved in its local transmission cycle.

The marked differences in prevalence among the three reptile species could indicate species-specific variation in exposure associated with their ecology. The almost universal infection detected in California kingsnakes exceeds the prevalence reported in most vertebrate paratenic hosts studied to date. Previous studies have suggested that snakes act as major accumulators of *A. cantonensis* because of their longevity and feeding habits [9]. In the Canary Islands, the California kingsnake preys on gastropods and endemic lizards such as *G. galloti* [16], whose role as a paratenic host has already been demonstrated [15]. This finding is consistent with the trophic biomagnification hypothesis widely proposed for *A. cantonensis*, whereby predators occupying higher trophic levels accumulate infective larvae through the consumption of previously infected prey [24]. Such a mechanism has been particularly well documented in predatory reptiles, including monitor lizards, in which high parasite burdens have been associated with their trophic position [10]. Therefore, California kingsnakes appears to be a key paratenic host within the Gran Canaria ecosystem and may play an important role in maintaining the transmission of *A. cantonensis* in invaded areas. Consequently, this invasive snake should be considered a valuable sentinel species for surveillance programs targeting this parasite of medical and veterinary importance.

In contrast, the moderate prevalence detected in Cuban green anoles suggests a more variable level of exposure, probably related to its predominantly insectivorous diet [27]. Nevertheless, the presence of the parasite in this species indicates that reptiles occupying lower trophic positions can also become incorporated into the transmission cycle, most likely through the accidental ingestion of gastropods or infected prey. This pattern is consistent with global studies showing that infection in paratenic hosts depends largely on the frequency of contact with intermediate hosts and the structure of local trophic networks [7]. The detection of *A. cantonensis* in Cuban green anoles is particularly relevant from an urban ecology perspective because this synanthropic species commonly inhabits gardens and irrigated green areas. Therefore, its infection therefore provides further evidence of active parasite circulation at the human–wildlife interface.

The absence of *A. cantonensis* DNA in veiled chameleons most likely reflects ecological differences in exposure rather than intrinsic resistance to infection. Several studies have shown that transmission of *A. cantonensis* is strongly influenced by environmental factors, particularly the availability of infected gastropods and interactions with rodent populations, resulting in considerable spatial heterogeneity in infection risk [28]. Consequently, the absence of positive individuals should be interpreted cautiosly. The lack of infection may be explained by the arboreal habits and strictly insectivorous diet of veiled chameleons [23,29], which greatly reduces contact with terrestrial gastropods and reinforces the importance of habitat stratification as a key determinant of parasite transmission [7].

From an epidemiological perspective, the significant differences in prevalence among species highlight the critical role of ecological and trophic factors in shaping infection dynamics. The high prevalence observed in California kingsnakes suggests greater cumulative exposure, whereas the lower prevalence in Cuban green anoles and the absence of infection in veiled chameleons probably reflect differences in ecological niches and interactions with intermediate hosts. Such interspecific variation has been previously reported for *A. cantonensis* and is generally associated with differences in ecology and feeding behavior [7,30]. Likewise, the absence of significant differences between sexes is consistent with previous studies indicating that infection probability in paratenic hosts depends primarily on trophic exposure rather than sex-related physiological factors. Diet, habitat use, and the availability of intermediate hosts have consistently been identified as the principal determinants of infection [7,30].

From a zoonotic perspective, these findings are particularly relevant because *A. cantonensis* is the etiological agent of human neuroangiostrongyliasis [4]. Human infection occurs through the ingestion of infective L3 larvae present in intermediate or paratenic hosts or through food and water contamination [31]. Although reptiles are not consumed in the Canary Islands, numerous human cases associated with reptile consumption have been reported, particularly in Asia and the Pacific region [10]. Beyond this direct transmission route, the presence of parasites in invasive reptiles demonstrates active environmental circulation, thereby increasing the indirect risk to humans through contaminated food, water, or infected intermediate hosts.

These findings have implications for biodiversity conservation and One Health surveillance in the Canary Islands. The detection of *A. cantonensis* in invasive reptiles indicates that these species are exposed to the parasite and may represent additional components of the local transmission network [24]. Given the ecological impact of invasive reptiles on endemic biodiversity, their role as paratenic hosts should be considered in future surveillance programs targeting *A. cantonensis* and other emerging zoonotic pathogens. Continued monitoring of invasive and native wildlife may help clarify the distribution, host range, and ecological dynamics of the parasite in newly colonized island ecosystems.

## 5. Conclusions

This study provides the first evidence that the invasive California kingsnake and Cuban green anole can act as natural paratenic hosts for *A. cantonensis* in the Canary Islands, and, to our knowledge, in Europe. These findings confirm the rapid and successful integration of this zoonotic nematode into the complex trophic network of the Canary Islands. The exceptionally high prevalence observed in California kingsnakes suggests that this species can act as a major parasite accumulator through trophic biomagnification, making it a valuable sentinel species for epidemiological monitoring. Furthermore, the detection of the parasite in synanthropic Cuban green anoles highlights active environmental circulation at the human–wildlife interface in urban and agricultural areas.

These findings expand the known host range of *A. cantonensis* and highlight the relevance of invasive wildlife in One Health surveillance. Further studies are needed to determine larval viability, trophic transmission, and the contribution of invasive reptiles to the maintenance and spatial distribution of the parasite in the Canary Islands.

This study has some limitations, such as the unequal sample sizes that may affect comparisons between reptile species. In addition, only liver tissue was analyzed, and therefore the presence or distribution of *A. cantonensis* in other tissues could not be assessed. The nested PCR assay demonstrates the presence of parasite DNA but does not confirm larval viability or infectivity. Further studies involving broader tissue sampling and investigation of larval viability and trophic interactions would help clarify the epidemiological and ecological significance of these findings.

## Figures and Tables

**Figure 1:**
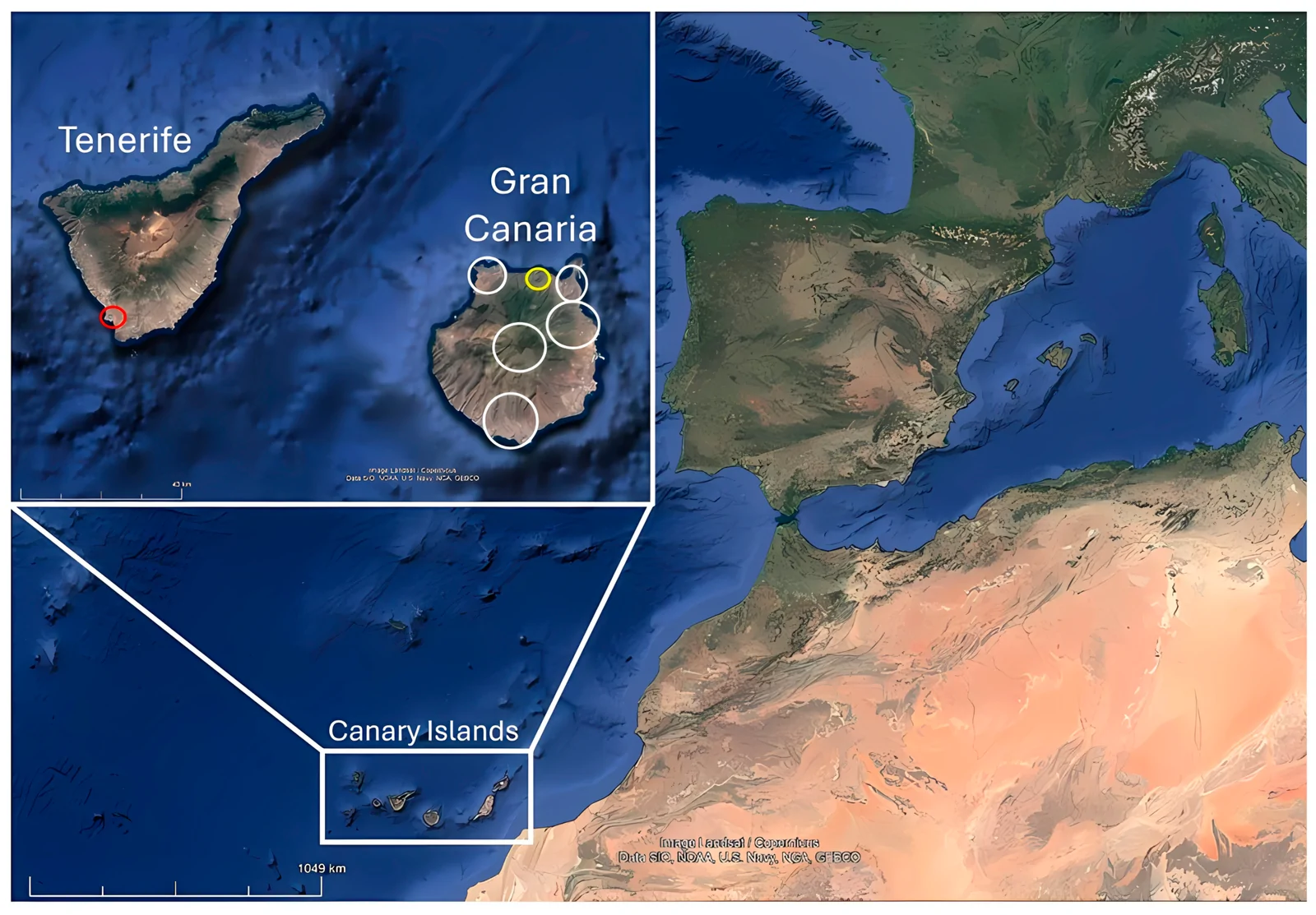
Location of Tenerife and Gran Canaria islands, where the specimens were collected. Image obtained from Google Earth Pro (v. 7.3.6.10441) and modified with Microsoft PowerPoint software (v. 2505). (White circles: *Lampropeltis californiae*; red circle: *Anolis porcatus*; yellow circle: *Chamaeleo calyptratus*).

**Figure 2:**
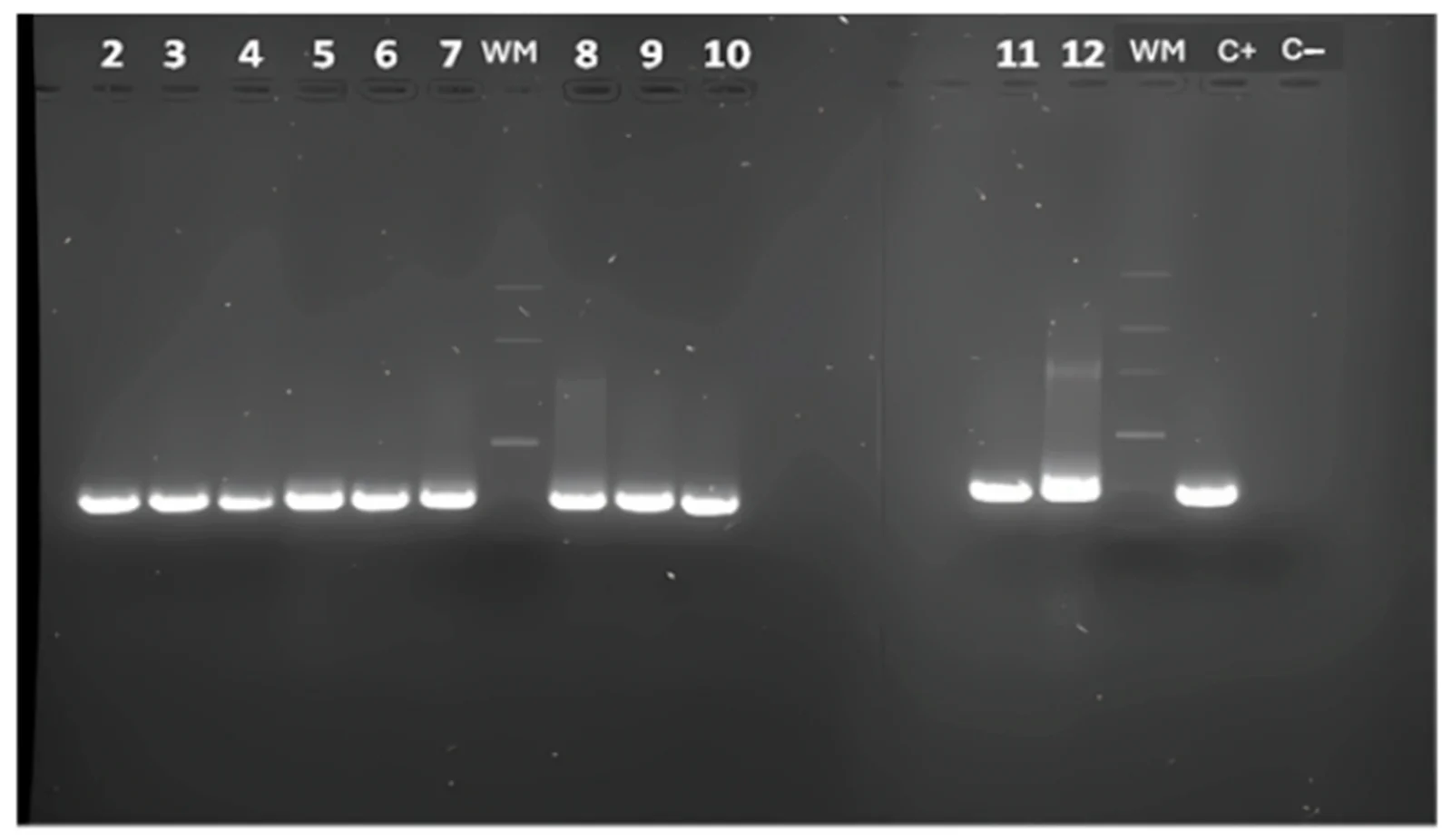
Results of the nested PCR assay for the detection of *Angiostrongylus cantonensis* (642 bp) in liver samples from *Lampropeltis californiae*. Lanes 2–7 and 8–12: liver samples; C+, positive control; C−, negative control; WM, 100–4000 bp molecular weight marker (VWR International, Haasrode, Belgium).

**Figure 3:**
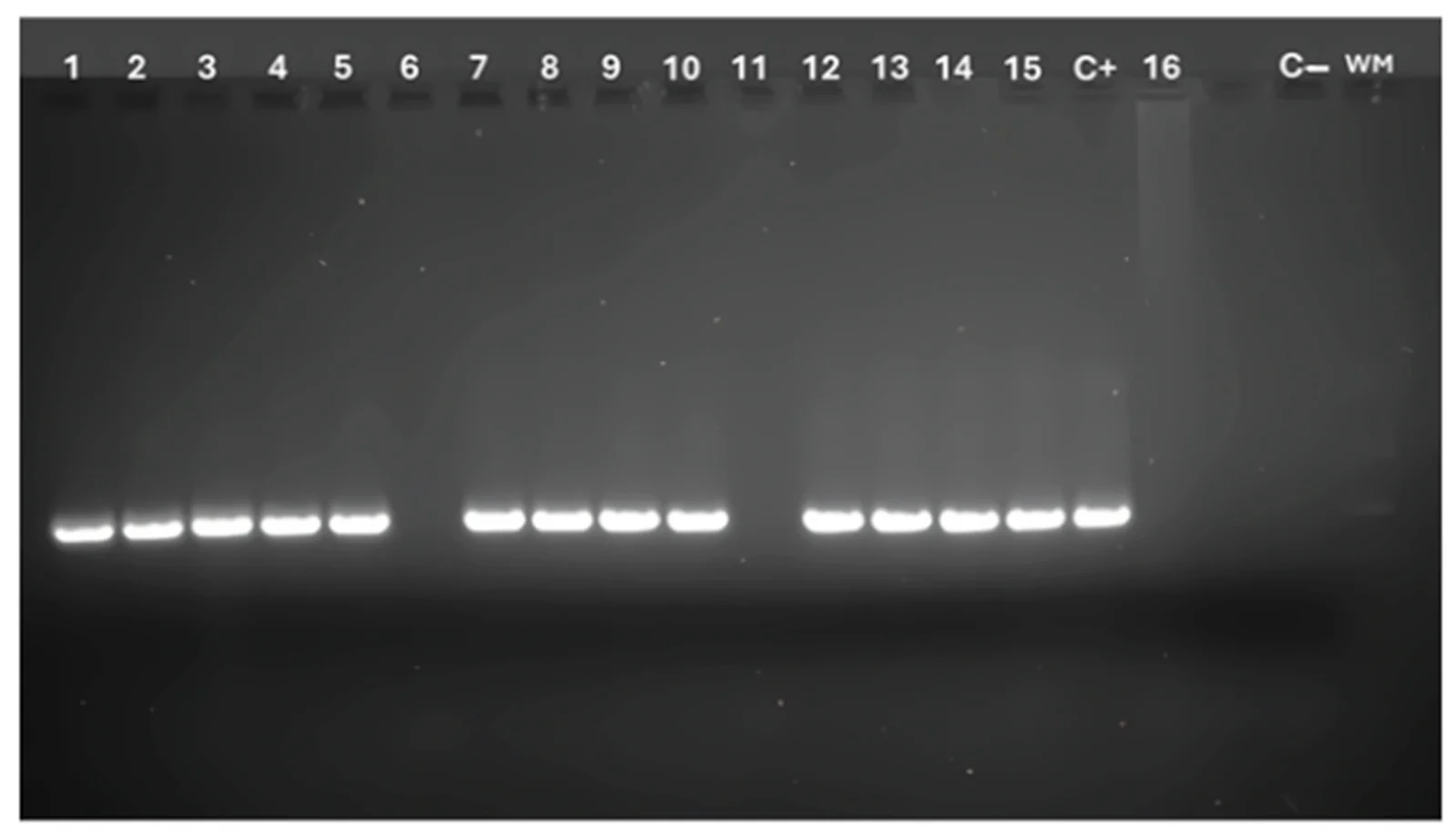
Results of the nested PCR assay for the detection of *Angiostrongylus cantonensis* (642 bp) in liver samples from *Anolis porcatus*. Lanes 1–16: liver samples; C+, positive control; C−, negative control; WM, 100–4000 bp molecular weight marker.

**Table 1:** Nucleotide sequences and target regions of the primers used for the nested PCR detection of *Angiostrongylus cantonensis*.

Primer Name	Sequence (5′–3′)	PCR Round	Target Gene/Region	Reference
AngioF1674	GTCGTAACAAGGTATCTGTAGGTG	1	Complete ITS1 * Region	Qvarnstrom et al. [23]
58SR4	TAGCTGCGTTTTTCATCGATA	1	Complete ITS1 * Region	Qvarnstrom et al. [23]
ITS1_CantoF3	AACAACTAGCATCATCTACGTC	2	Specific ITS1 * *Angiostrongulys cantonensis* region	Izquierdo-Rodríguez et al. [24]
ITS1_CantoR1	CATCCTGTGTATCTCGTTCC	2	Specific ITS1 * *Angiostrongulys cantonensis* region	Izquierdo-Rodríguez et al. [24]

* Internal transcribed spacer 1 (ITS1).

**Table 2:** Prevalence of *Angiostrongylus cantonensis* in the liver of *Lampropeltis californiae* from Gran Canaria and *Anolis porcatus* from Tenerife (Canary Islands, Spain).

Host Species	Sex	Positive/Total	Prevalence (%) (95% CI)
*Lampropeltis californiae*	Males	32/34	94.1 (80.3–99.3)
	Females	11/12	91.7 (61.5–99.8)
	Undetermined	5/5	100 (47.8–100)
*Anolis porcatus*	Males	6/25	24.0 (9.3–45.1)
	Females	11/45	24.4 (12.8–39.5)
	Juveniles	0/4	0 (0.0–60.2)

**Table 3:** Prevalence of *Angiostrongylus cantonensis* in the liver of the three reptile species examined.

Species	Sex	Positive/Total	Prevalence (%) (95% CI)
*Lampropeltis californiae*		48/51	94.1 (83.4–98.7)
	Male	32/34	93.9 (83.4–98.7)
	Female	11/12	92.3 (63.9–99.8)
*Anolis porcatus*		16/74	21.6 (12.8–32.7)
	Male	6/25	24.0 (9.3–45.1)
	Female	11/45	24.4 (12.8–39.5)
*Chamaeleo calyptratus*		0/42	0 (0.0–0.8)
	Male	0/14	0 (0.0–23.1)
	Female	0/22	0 (0.0–15.4)

## Data Availability

The original contributions presented in the study are included in the article. The nucleotide sequences generated are openly available in the GenBank database under accession numbers PX657145–PX657161.
